# Supplementary material for: Mutation of the peptide-regulated transcription factor ComR for amidated peptide specificity and heterologous function in Lactiplantibacillus plantarum WCFS1
Source: Microbiol Spectr. 2024 Apr 30;12(6):e00517-24. doi: 10.1128/spectrum.00517-24 (PMC11237612; doi:10.1128/spectrum.00517-24)
Supplement: Supplemental material — Table S1 and S2; Fig. S1 to S3. [file spectrum.00517-24-s0001.pdf]

Supplemental Data For:

Mutation of the peptide-regulated transcription factor ComR for amidated peptide specificity and heterologous function in *Lactiplantibacillus plantarum* WCFS1.

Figure S1: Replicate-in-time data for Figure 1.

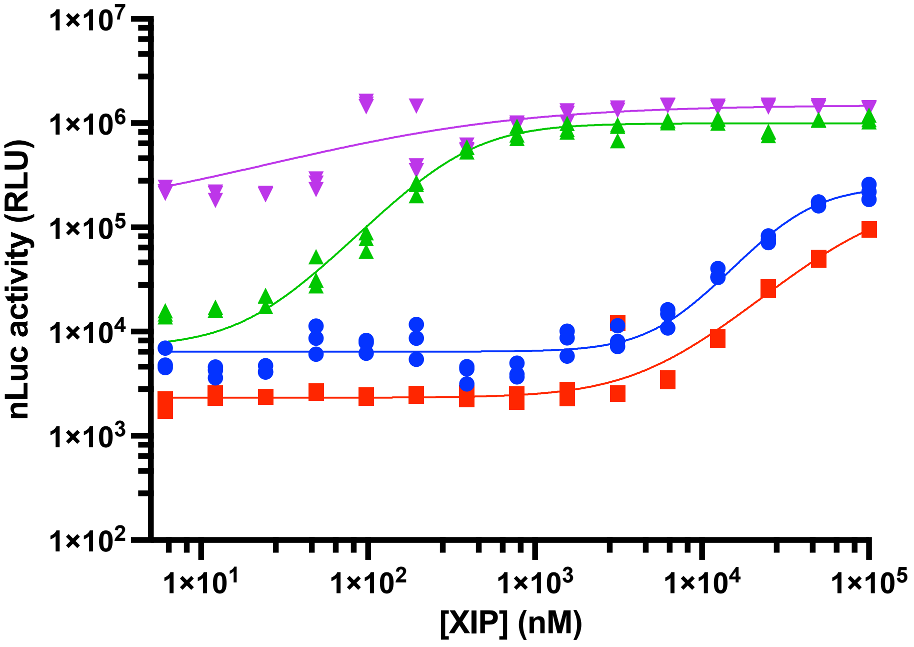

Figure S1: Identical experiment as that presented in Figure 1, performed on a different data.

Figure S2: Average root mean square deviation of the terminal residue (Leu24) of the amidated XIP peptide while in the ComR pocket.

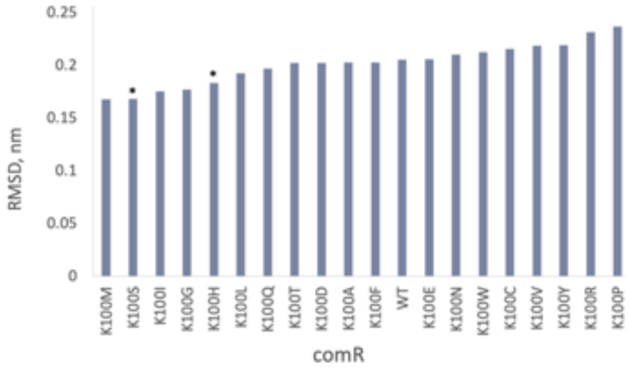

Figure S2: We performed molecular dynamics (MD) simulations of the amidated XIP peptide in the ComR pocket for wild type (WT) K100 ComR and all 20 single AA mutations at that position. To quantify the favorability of the mutations to accommodate the XIP peptide, we calculated the average root mean square deviation (RMSD) of the terminal residue (Leu24) of the XIP peptide, which is in direct contact with K100. Fig X shows the RMSD for all the ComR mutants ranked from least to the most. Consistent with the minimal RMSD observed for K100S and K100H, they show favorable binding to the amidated XIP.

Figure S3: 5x Mut of ComR vs XIP_{th} and XIP_{vest} in *E. coli*

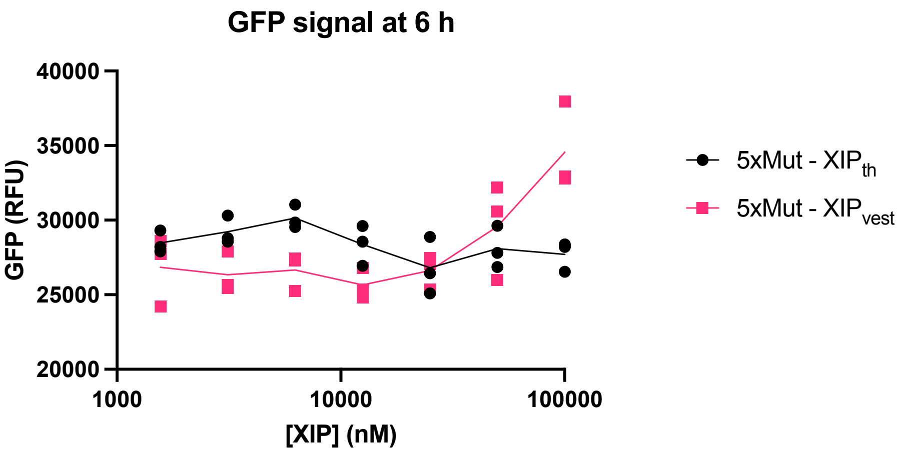

Figure S3: The GFP response to the indicated concentrations of XIP from *S. thermophilus* or *S. vestibularis* are shown for ComR mutated with five mutations (R92G, V205A, S248G, S289K, I290T) expressed in *E. coli.* GFP signal after 6 hours of incubation is shown for triplicate experiments with a connecting line bridging the means.

Table S1: Strains and plasmids developed in the current work.

**Strain / Plasmid**	**Parent**	**Description**
v1	WCFS1 with an RBS and ComR CDS inserted downstream of the *rpsU* gene. Inserted downstream of the ComR is a transcription terminator, promoter and secreted nLuc CDS. Finally, an erythromycin resistance cassette is inserted as well.	RBS from *ldh* (*S. sanguinis*) , Terminator from *rrnC* (*E. coli*) and promoter from *comS S. thermophilus.*
v2		RBS from *ldh* (S. sanguinis) , Terminator is L3S2P21 (synthetic) and promoter from STER_1655 S. thermophilus.
v3		Partial RBS from *ldh* (S. sanguinis) , Terminator is L3S2P21 (synthetic) and promoter from STER_1655 S. thermophilus.
v4		no RBS, Terminator is L3S2P21 (synthetic) and promoter from STER_1655 S. thermophilus.
pAC-J23107*-ComR	pACYC184 plasmid expressing ComR constitutively and GFP expressed via a ComR responsive promoter.	ComR expressed via a strong RBS and a point mutant of J23107.
pAC-J23116-ComR		ComR expressed via a strong RBS and promoter J23116.
pTRK-xyl-ComR-v2	pTRKH2	ComR expressed from a xylose inducible cassette. nLuc expressed in the same format as strain v2 above.

Table S2: Full Plasmid and component sequences:

**Genetic Component**	**Sequence**

ComR	ttgaacttaaaagacagcattggactaagaatcaaaactgagcgtgaacgccaacagatgtcacgtgaagtgctatgtttagatggtg<br>cggaattgactgttcgccagttaattcgtattgaaaagggggagtctctcccgtctttagatagattatcgtatattgctaaacgtttaggaa<br>aaagtatgacagagttattggatcaagacaatattaccattcctgacgaatattatgaaatgaagaatcgtttgattaagtttccaacgta<br>cagaaaccctgacagaataaagtctaaacttactttgattgaggaagtctatgagaaatttttgatattcttccagaagaagaattattaa<br>ctctagacattctcgaaaatatattgagttttactagctgggaggagagtccaaaagttgaggagatatatgaagacttgtttgaacaagt<br>caaaaggaagaggaaattctcaactaacgatttattagtcattgactattatttctttcatctttatgggagaaaacagtatgacaaaaaa<br>ctatttgaaagaattataaagagagtattaaatcaggaaatttggacagatgatgtttacaatattgttttatttaatgatttgatggctattgct<br>gctttaaagatttttcacaattccttctcagacttcttaacagttgtggataaagccttagctgtcatagaaaaatcacaattatatagctaca<br>agcctagtgtttttgtacttaaggctaaatatgaacttctgcataaagaaaacaagaaagaggctgcagagaattatgataaggccata<br>atgtttgcttccgttttggaagactcggttttagaggaaagtataaaggcaggaaaattggcagatggtttatag
Secreted nLuc	atgaaaaaatttaactttaaaaccatgttgctattagttttggctagttgtgtcttcggggtcgtcgttaacgtgactactagtcttggaccaca<br>aaccgcaatcaccgcccaggcctccaagatggttttcacgttagaagatttcgttggtgactggcgtcaaactgctggttataatttggat<br>caagttttagaacagggtggtgtttcaagtttatttcaaaatttgggtgtcagtgttacgccaattcaacggattgttttaagtggtgaaaatg<br>gtttaaagatcgatattcatgtcatcattccatatgaaggtttatcaggtgaccaaatgggtcaaattgaaaaaattttcaaggtcgtttacc<br>cagttgatgatcatcattttaaagttatcttgcactacggtacgttagttattgatggtgttacgccaaatatgattgattattttggtcggccata<br>tgaaggcattgcagtttttgatggtaaaaagattacggttactggtacgttatggaatggtaataagattattgacgaacggttaattaacc<br>cagatggtagtttattattccgtgttacgattaatggtgttactggttggcgtttatgtgaacgtattttagcttaa
L3S2P21	CTCGGTACCAAATTCCAGAAAAGAGGCCTCCCGAAAGGGGGGCCTTTTTTCGTTTTGGTCC
p1655	AAATCGAGTAGTGACATTTATGTCACTACTTTTTTGTTGGCTACCACCCTATAATAAAATCA
pAC-J23107*-ComR	TTGAGATCGTTTTGGTCTGCGCGTAATCTCTTGCTCTGAAAACGAAAAAACCGCCTTGCAGG<br>GCGGTTTTTCGAAGGTTCTCTGAGCTACCAACTCTTTGAACCGAGGTAACTGGCTTGGAGG<br>AGCGCAGTCACCAAAACTTGTCCTTTCAGTTTAGCCTTAACCGGCGCATGACTTCAAGACTA<br>ACTCCTCTAAATCAATTACCAGTGGCTGCTGCCAGTGGTGCTTTTGCATGTCTTTCCGGGTT<br>GGACTCAAGACGATAGTTACCGGATAAGGCGCAGCGGTCGGACTGAACGGGGGGTTCGTG<br>CATACAGTCCAGCTTGGAGCGAACTGCCTACCCGGAACTGAGTGTCAGGCGTGGAATGAG<br>ACAAACGCGGCCATAACAGCGGAATGACACCGGTAAACCGAAAGGCAGGAACAGGAGAGC<br>GCACGAGGGAGCCGCCAGGGGGAAACGCCTGGTATCTTTATAGTCCTGTCGGGTTTCGCC<br>ACCACTGATTTGAGCGTCAGATTTCGTGATGCTTGTCAGGGGGGCGGAGCCTATGGAAAAA<br>CGGCTTTGCCGCGGCCCTCTCACTTCCCTGTTAAGTATCTTCCTGGCATCTTCCAGGAAATC<br>TCCGCCCCGTTCGTAAGCCATTTCCGCTCGCCGCAGTCGAACGACCGAGCGTAGCGAGTC<br>AGTGAGCGAGGAAGCGGAATATATCCTtttacggctagctcagccctaggtataatgctagcattaaagaggagaaa<br>tactagAtgaacttaaaagacagcattggactaagaatcaaaactgagcgtgaacgccaacagatgtcacgtgaagtgctatgttta<br>gatggtgcggaattgactgttcgccagttaattcgtattgaaaagggggagtctctcccgtctttagatagattatcgtatattgctaaacgtt<br>taggaaaaagtatgacagagttattggatcaagacaatattaccattcctgacgaatattatgaaatgaagaatcgtttgattaagtttcc<br>aacgtacagaaaccctgacagaataaagtctaaacttactttgattgaggaagtctatgagaaatttttgatattcttccagaagaaga<br>attattaactctagacattctcgaaaatatattgagttttactagctgggaggagagtccaaaagttgaggagatatatgaagacttgtttg<br>aacaagtcaaaaggaagaggaaattctcaactaacgatttattagtcattgactattatttctttcatctttatgggagaaaacagtatgac<br>aaaaaactatttgaaagaattataaagagagtattaaatcaggaaatttggacagatgatgtttacaatattgttttatttaatgatttgatgg<br>ctattgctgctttaaagatttttcacaattccttctcagacttcttaacagttgtggataaagccttagctgtcatagaaaaatcacaattatat<br>agctacaagcctagtgtttttgtacttaaggctaaatatgaacttctgcataaagaaaacaagaaagaggctgcagagaattatgataa<br>ggccataatgtttgcttccgttttggaagactcggttttagaggaaagtataaaggcaggaaaattggcagatggtttatagcagataaa<br>aaaaatccttagctttcgctaaggatgatttctaaatggtggtgacataaatgtcactactttttttagggttaaatatgtactatagaaatata<br>aataatagttaaaaggagttatattatgagtaaaggagaagaacttttcactggagttgtcccaattcttgttgaattagatggtgatgttaa<br>tgggcacaaattttctgtcagtggagagggtgaaggtgatgcaacatacggaaaacttacccttaaatttatttgcactactggaaaact<br>acctgttccatggccaacacttgtcactactttcggttatggtgttcaatgctttgcgagatacccagatcatatgaaacagcatgactttttc<br>aagagtgccatgcccgaaggttatgtacaggaaagaactatatttttcaaagatgacgggaactacaagacacgtgctgaagtcaag<br>tttgaaggtgataccttgttaatagaatcgagttaaaaggtattgattttaaagaagatggaaacattcttggacacaaattggaataca<br>actataactcacacaatgtatacatcatggcagacaaacaaaagaatggaatcaaagttaacttcaaaattagacacaacattgaag<br>atggaagcgttcaactagcagaccattatcaacaaaatactccaattggcgatggccctgtccttttaccagacaaccattacctgtcca<br>cacaatctgccctttcgaaagatcccaacgaaaagagagaccacatggtccttcttgagtttgtaacagctgctgggattacacatggc<br>atggatgatctctacaaataaTCAGGTCGAGGTGGCCCGGCTCCATGCACCGCGACGCAACGCGGG<br>GAGGCAGACAAGGTATAGGGCGGCGCCTACAATCCATGCCAACCCGTTCCATGTGCTCGCC

	GAGGCGGCATAAATCGCCGTGACGATCAGCGGTCCAATGATCGAAGTTAGGCTGGTAAGAG<br>CCGCGAGCGATCCTTGAAGCTGTCCCTGATGGTCGTCATCTACCTGCCTGGACAGCATGGC<br>CTGCAACGCGGGCATCCCGATGCCGCCGGAAGCGAGAAGAATCATAATGGGGAAGGCCAT<br>CCAGCCTCGCGTCGCGAACGCCAGCAAGACGTAGCCCAGCGCGTCGGCCGCCATGCCGG<br>CGATAATGGCCTGCTTCTCGCCGAAACGTTTGGTGGCGGGACCAGTGACGAAGGCTTGAG<br>CGAGGGCGTGCAAGATTCCGAATACCGCAAGCGACAGGCCGATCATCGTCGCGCTCCAGC<br>GAAAGCGGTCCTCGCCGAAAATGACCCAGAGCGCTGCCGGCACCTGTCCTACGAGTTGCA<br>TGATAAAGAAGACAGTCATAAGTGCGGCGACGATAGTCATGCCCCGCGCCCACCGGAAGGA<br>GCTGACTGGGTTGAAGGCTCTCAAGGGCATCGGTCGACGCTCTCCCTTATGCGACTCCTG<br>CATTAGGAAGCAGCCCAGTAGTAGGTTGAGGCCGTTGAGCACCGCCGCCGCAAGGAATGG<br>TGCATGCAAGGAGATGGCGCCCAACAGTCCCCCGGCCACGGGGCCTGCCACCATACCCAC<br>GCCGAAACAAGCGCTCATGAGCCCGAAGTGGCGAGCCCGATCTTCCCCATCGGTGATGTC<br>GGCGATATAGGCGCCAGCAACCGCACCTGTGGCGCCGGTGATGCCGGCCACGATGCGTCC<br>GGCGTAGAGGATCCACAGGACGGGTGTGGTCGCCATGATCGCGTAGTCGATAGTGGCTCC<br>AAGTAGCGAAGCGAGCAGGACTGGGCGGCGGCCAAAGCGGTCGGACAGTGCTCCGAGAA<br>CGGGTGCGCATAGAAATTGCATCAACGCATATAGCGCTAGCAGCACGCCATAGTGACTGGC<br>GATGCTGTCGGAATGGACGATATCCCGCAAGAGGCCCGGCAGTACCGGCATAACCAAGCCT<br>ATGCCTACAGCATCCAGGGTGACGGTGCCGAGGATGACGATGAGCGCATTGTTAGATTTCA<br>TACACGGTGCCTGACTGCGTTAGCAATTTAACTGTGATAAACTACCGCATTAAAGCTTATCGA<br>TGATAAGCTGTCAAACATGAGAATTACAACTTATATCGTATGGGGCTGACTTCAGGTGCTACA<br>TTTGAAGAGATAAATTGCACTGAAATCTAGAAATATTTTATCTGATTAATAAGATGATCTTC

pTRK-xyl-ComR-v2	tttccataggctccgcccccctgacaagcatcacgaaatctgacgctcaaatcagtggtggcgaaacccgacaggactataaagata<br>ccaggcgtttccccctggcggctccctcgtgcgctctcctgttcctgcctttcggtttaccggtgtcattccgctgttatggccgcgtttgtctca<br>ttccacgcctgacactcagttccgggtaggcagttcgctccaagctggactgtatgcacgaaccccccgttcagtccgaccgctgcgcc<br>ttatccggtaactatcgtcttgagtccaacccggaaagacatgcaaaagcaccactggcagcagccactggtaattgatttagaggag<br>ttagtcttgaagtcatgcgccggttaaggctaaactgaaaggacaagttttggtgactgcgctcctccaagccagttacctcggttcaaa<br>gagttggtagctcagagaaccttcgaaaaaccgccctgcaaggcggttttttcgttttcagagcaagagattacgcgcagaccaaaac<br>gatctcaagaagatcatcttattaatcagataaaatatttctagccaatacgcaaaccgcctctccccgcgcgttggccgattcattaatg<br>cagctggcacgacaggtttcccgactggaaagcgggcagtgagcgcaacgcaattaatgtgagttagctcactcattaggcacccca<br>ggctttacactttatgcttccggctcgtatgttgtgtggaattgtgagcggataacaatttcacacaggaaacagctctaacttataggggt<br>aacacttaaaaaagaatcaataacgatagaaaccgctcctaaagcaggtgcatttttcctaacgaagaaggcaatagttcacatttat<br>tgtctaaatgagaatggactctagaagaaacttcgtttttaatcgtatttaaaacaatgggatgagattcaattatatgatttctcaagataa<br>cagcttctatatcaaatgtattaaggatattggttaatccaattccgatataaaagccaaagttttgaagtgcatttaacatttctacatcatttt<br>tatttgcgcgttccacaatctcttttcgagaaatattcttttcttctttagagagcgaagccagtaacgctttttcagaagcatataattcccaa<br>cagcctcgatttccacagctgcatttgggtccattaaaatctatcgtcatatgacccatttccccagaaaaaccctgaacacctttataca<br>attcgttgttaataacaagtccagttccaattccgatattaatactgatgtaaacgatgttttcatagttttttgtcataccaaatactttttcacc<br>gtatgctcctgcattagcttcattttcaacaaaaaccggaacattaaactcactctcaattaaaaactgcaaatctttgatattccaatttaa<br>gttaggcatgaaaataatttgctgatgacgatctacaaggcctggaacacaaattcctattccgactagaccataaggggactcaggc<br>atatgggttacaaaaccatgaataagtgcaaataaaatctcttttacttcactagcggaagaactagacaagtcagaagtcttctcgag<br>aataatatttccttctaagtcggttagaattccgttaagatagtcgactcctatatcaataccaatcgagtagcctgcattcttattaaaaaca<br>agcattacaggtcttctgccgcctctagattgccctgccccaatttcaaaaataaaatctttttcaagcagtgtatttacttgagaggagac<br>agtagacttgtttaatcctgtaatctcagagagagttgccctggagacaggggagttcttcaaaatttcatctaatattaatttttgattcattttt<br>tttactaaagcttgatctgcaatttgaataataaccactcctttgtttatcctcctctagtctaaaactcttttatattatatcacaaataaggctct<br>ttttcagctattctactatagttttccgctgagaaaggtaaatattagtgactttcttaacaaaaagtgttagaatgagataaagttagtttattg<br>gataaacaaactaactcaattaagatagttgatggataaacttgttcacttaaatcaaaggaggtaaatttgaacttaaaagacagcatt<br>ggactaagaatcaaaactgagcgtgaacgccaacagatgtcacgtgaagtgctatgtttagatggtgcggaattgactgttcgccagtt<br>aattcgtattgaaaagggggagtctctcccgtctttagatagattatcgtatattgctaaacgtttaggaaaaagtatgacagagttattgg<br>atcaagacaatattaccattcctgacgaatattatgaaatgaagaatcgtttgattaagtttccaacgtacagaaaccctgacagaataa<br>agtctaaacttactttgattgaggaagtctatgagaaatttttgatattcttccagaagaagaattattaactctagacattctcgaaaatat<br>attgagttttactagctgggaggagagtccaaaagttgaggagatatatgaagacttgtttgaacaagtcaaaaggaagaggaaattc<br>tcaactaacgatttattagtcattgactattatttctttcatctttatgggagaaaacagtatgacaaaaaaactatttgaaagaattataaaga<br>gagtattaaatcaggaaatttggacagatgatgtttacaatattgttttatttaatgatttgatggctattgctgctttaaagatttttcacaattcc<br>ttctcagacttcttaacagttgtggataaagccttagctgtcatagaaaaatcacaattatatagctacaagcctagtgtttttgtacttaagg<br>ctaaatatgaacttctgcataaagaaaacaagaaagaggctgcagagaattatgataaggccataatgtttgcttccgttttggaagac<br>tcggttttagaggaaagtataaaggcaggaaaattggcagatggtttatagCTCGGTACCAAATTCCAGAAAAGAGG<br>CCTCCCGAAAGGGGGGCCTTTTTTCGTTTTGGTCCtgatttctAAATCGAGTAGTGACATTTATGT<br>CACTACTTTTTTGTTGGCTACCACCCTATAATAAAATCAtaaataatagttaaaaggagttatattatgaaaaaa<br>tttaactttaaaaccatgttgctattagttttggctagttgtgtcttcggggtcgtcgttaacgtgactactagtcttggaccacaaaccgcaat<br>caccgcccaggcctccaagatggttttcacgttagaagatttcgttggtgactggcgtcaaactgctggttataattggatcaagttttaga<br>acagggtggtgtttcaagtttatttcaaaatttgggtgtcagtgttacgccaattcaacggattgttttaagtggtgaaaatggtttaaagatc<br>gatattcatgtcatcattccatatgaaggtttatcaggtgaccaaatgggtcaaattgaaaaaattttcaaggtcgtttacccagttgatgat<br>catcattttaaagttatcttgcactacggtacgttagttattgatggtgttacgccaaatatgattgattattttggtcggccatatgaaggcatt<br>gcagtttttgatggtaaaaagattacggttactggtacgttatggaatggtaataagattattgacgaacggttaattaacccagatggtag<br>tttattattccgtgttacgattaatggtgttactggttggcgtttatgtgaacgtattttagcttaaaatcgatacgattttgaagtggcaacagat<br>aaaaaaaagcagtttaaaattgttgctgaactttaaaacaagcaaatacaatcattgtcgcaacagatagcgacagagaaggcga<br>aaacattgcctggtcgatcattcataaagcaaatgccttttctaaagataaaacgtataaaagactatggatcaatagtttagaaaaag<br>atgtgatccgtagcggttttcaaaatttgcaaccaggaatgaattactatccctttatcaagaagcgcacaaaaagaaaaacgaaatg<br>atacaccaatcagtgcaaaaaaagaagttcctattctctagaaagtataggaacttcgatataatgggagataagacggttcgtgttcgt<br>gctgacttgcaccatatcataaaaatcgaaacagcaaagaatggcggaaacgtaaaagaagttatggaaataagacttagaagca<br>aacttaagagtgtgttgatagtgcagtatcttaaaattttgtataataggaattgaagttaaattagatgctaaaaatttgtaattaagaagg<br>agtgattacatgaacaaaaatataaaatattctcaaaactttttaacgagtgaaaaagtactcaaccaaataataaaacaattgaattta<br>aaagaaaccgataccgtttacgaaattggaacaggtaaagggcatttaacgacgaaactggctaaaataagtaaacaggtaacgtc<br>tattgaattagacagtcatctattcaacttatcgtcagaaaaattaaaactgaatactcgtgtcactttaattcaccaagatattctacagttt<br>caattccctaacaaacagaggtataaaattgttgggagtattccttaccatttaagcacacaaattattaaaaaagtggtttttgaaagcc<br>atgcgtctgacatctatctgattgttgaagaaggattctacaagcgtaccttggatattcaccgaacactagggttgctcttgcacactcaa<br>gtctcgattcagcaattgcttaagctgccagcggaatgctttcatcctaaaccaaaagtaaacagtgtcttaataaaacttacccgccat<br>accacagatgttccagataaatattggaagctatatacgtactttgtttcaaaatgggtcaatcgagaatatcgtcaactgtttactaaaaa

	tcagtttcatcaagcaatgaaacacgccaaagtaaacaatttaagtaccgttacttatgagcaagtattgtctatttttaatagttatctattat<br>ttaacgggaggaaataagaagttcctattccgaagttcctattctctagaaagtataggaacttcttctatgagtcgcttttgtaaatttggaa<br>agttacacgttactaaagggaatgtagataaattattaggtatactactgacagcttccaaggagctaaagaggtccctagcgctcttat<br>catggggaagctcggatcatatgcaagacaaaataaactcgcaacagcacttggagaaatgggacgaatcgagaaaaccctcttt<br>acgctggattacatatctaataaagccgtaaggagacgggttcaaaaaggtttaaataaaggagaagcaatcaatgcattagctaga<br>attatatttttggacaacgtggagaatttagagaacgtgctctccaagaccagttacaaagagctagtgcactaaacataattattaacg<br>ctataagtgtgtggaacactgtatatatggaaaaagccgtagaagaattaaaagcaagaggagaatttagagaagatttaatgccat<br>atgcgtggccgttaggatgggaacatatcaattttcttggagaatacaaatttgaaggattacatgacactgggcaaatgaatttacgtcc<br>tttacgtataaaagagccgttttattcttaatataacggctctttttatagaaaaaatccttagcgtggttttttccgaaatgctggcggtaccc<br>caagaattagaaatgagtagatcaaattattcacgaatagaatcaggaaaatcagatccaaccataaaaacactagaacaaattgc<br>aaagttaactaactcaacgctagtagtggatttaatcccaaatgagccaacagaaccagaaccagaaacagaatcagaacaagta<br>acattggatttagaaatggaagaagaaaaaagcaatgacttcgtgtgaataatgcacgaaatcgttgcttattttttttaaaagcggtata<br>ctagatataacgaaacaacgaactgaatagaaacgaaaaaagagccatgacacatttataaaatgtttgacgacattttataaatgc<br>atagcccgataagattgccaaaccaacgcttatcagttagtcagatgaactcttccctcgtaagaagttatttaattaactttgtttgaagac<br>ggtatataaccgtactatcattatatagggaaatcagagagttttcaagtatctaagctactgaatttaagaattgttaagcaatcaatcgg<br>aaatcgtttgattgcttttttgtattcatttatagaaggtggagtttgtatgaatcatgatgaatgtaaaacttatataaaaaatagtttattgga<br>gataagaaaattagcaaatatctatacactagaaacgtttaagaaagagttagaaaagagaaatatctacttagaaacaaaatcaga<br>taagtatttttcttcggaggggaagattatatatataagttaatagaaaataacaaaataatttattcgattagtggaaaaaaattgactta<br>taaaggaaaaaaatctttttcaaaacatgcaatattgaaacagttgaatgaaaaagcaaaccaagttaattaaacaacctattttatag<br>gatttataggaaaggagaacagctgaatgaatatcccttttgttgtagaaactgtgcttcatgacggcttgttaaagtacaaatttaaaaat<br>agtaaaattcgctcaatcactaccaagccaggtaaaagcaaaggggctatttttgcgtatcgctcaaaatcaagcatgattggcggtcg<br>tggtgttgttctgacttccgaggaagcgattcaagaaaatcaagatacatttacacattggacacccaacgtttatcgttatggaacgtat<br>gcagacgaaaaccgttcatacacgaaaggacattctgaaaacaatttaagacaaatcaataccttctttattgattttgatattcacacg<br>gcaaaagaaactatttcagcaagcgatattttaacaaccgctattgatttaggttttatgcctactatgattatcaaatctgataaaggttatc<br>aagcatattttgttttagaaacgccagtctatgtgacttcaaaatcagaatttaaatctgtcaaagcagccaaaataatttcgcaaaatatc<br>cgagaatattttggaaagtctttgccagttgatctaacgtgtaatcattttggtattgctcgcataccaagaacggacaatgtagaattttttg<br>atcctaattaccgttattctttcaaagaatggcaagattggtctttcaaacaaacagataataagggctttactcgttcaagtctaacggtttt<br>aagcggtacagaaggcaaaaaacaagtagatgaaccctggtttaatctcttattgcacgaaacgaaattttcaggagaaaagggttt<br>aatagggcgtaataacgtcatgtttaccctctctttagcctactttagttcaggctattcaatcgaaacgtgcgaatataatatgtttgagttta<br>ataatcgattagatcaacccttagaagaaaaagaagtaatcaaaattgttagaagtgcctattcagaaaactatcaaggggctaatag<br>ggaatacattaccattctttgcaaagcttgggtatcaagtgatttaaccagtaaagatttatttgtccgtcaagggtggtttaaattcaagaa<br>aaaaagaagcgaacgtcaacgtgttcatttgtcagaatggaaagaagatttaatggcttatattagcgaaaaaagcgatgtatacaag<br>ccttatttagtgacgaccaaaaaagagattagagaagtgctaggcattcctgaacggacattagataaattgctgaaggtactgaagg<br>cgaatcaggaaattttctttaagattaaaccaggaagaaatggtggcattcaacttgctagtgttaaatcattgttgctatcgatcattaaa<br>gtaaaaaaagaagaaaaagaaagctatataaaggcgctgacaaattcttttgacttagagcatacattcattcaagagactttaaaca<br>agctagcagaacgccctaaaacggacacacaactcgatttgtttagctatgatacaggctgaaaataaaacccgcactatgccatta<br>catttatatctatgatacgtgtttgtttttctttgctgtttagcgaatgattagcagaaatatacagagtaagattttaattaattattaggggga<br>gaaggagagagtagcccgaaaacttttagttggcttggactgaacgaagtgagggaaaggctactaaaacgtcgaggggcagtga<br>gagcgaagcgaacacttgatttttaattttctatcttttataggtcattagagtatacttatttgtcctataaactatttagcagcataatagattt<br>attgaataggtcatttaagttgagcatattagaggaggaaaatcttggagaaatatttgaagaacccgattacatggattggattagttctt<br>gtggttacgtggtttttaactaaaagtagtgaatttttgatttttggtgtgtgtgtcttgttgttagtatttgctagtcaaagtgattaaatagaattc<br>cggatgagcattcatcaggcgggcaagaatgtgaataaaggccggataaaacttgtgcttatttttctttacggtctttaaaaaggccgta<br>atatccagctgaacggtctggttataggtacattgagcaactgactgaaatgcctcaaaatgttctttacgatgccattgggatatatcaa<br>cggtggtatatccagtgatttttttctccattttagcttccttagctcctgaaaatctcgataactcaaaaaatacgcccggtagtgatcttattt<br>cattatggtgaaagttggaacctcttacgtgccgatcaacgtctcattttcgccaaaagttggcccagggcttcccggtatcaacaggga<br>caccaggatttatttattctgcgaagtgatcttccgtcacaggtatttattcggcgcaaagtgcgtcgggtgatgctgccaacttactgattta<br>gtgtatgatggtgtttttgaggtgctccagtggcttctgtttctatcagctgtccctcctgttcagctactgacggggtggtgcgtaacggcaa<br>aagcaccgccggacatcagcgctagcggagtgtatactggcttactatgttggcactgatgagggtgtcagtgaagtgcttcatgtggc<br>aggagaaaaaaggctgcaccggtgcgtcagcagaatatgtgatacaggatatattccgcttcctcgctcactgactcgctacgctcgg<br>tcgttcgactgcggcgagcggaaatggcttacgaacggggcggagatttcctggaagatgccaggaagatacttaacagggaagtg<br>agagggccgcggcaaagccgtt
